# Photodynamic therapy with a novel photosensitizer inhibits DSS-induced ulcerative colitis in rats via the NF-κB signaling pathway

**DOI:** 10.3389/fphar.2024.1539363

**Published:** 2025-01-08

**Authors:** Yumei Rong, Minghui Zhu, Nan Wang, Feiyu Zhang, Tianjun Liu

**Affiliations:** ^1^ The Third Central Hospital of Tianjin, Tianjin, China; ^2^ Tianjin Key Laboratory of Extracorporeal Life Support for Critical Diseases, Tianjin, China; ^3^ Artificial Cell Engineering Technology Research Center, Tianjin, China; ^4^ Tianjin Institute of Hepatobiliary Disease, Tianjin, China; ^5^ Tianjin Key Laboratory of Biomedical Material, Institute of Biomedical Engineering, Chinese Academy of Medical Sciences and Peking Union Medical College, Tianjin, China

**Keywords:** ulcerative colitis, DSS, oxidative stress, NF-κB signaling pathway, gut microbiota

## Abstract

**Introduction:**

Ulcerative colitis (UC) is an inflammatory bowel disease characterized by inflammation and ulceration of the digestive tract.

**Methods:**

Photodynamic therapy (PDT) with a novel photosensitizer LD_4_ was used to treat UC rat models to explore the therapeutic effect and mechanism of LD_4_-PDT on UC. 16S ribosomal RNA was used to detect the composition of Gut microbiota.

**Results:**

Our findings indicate that LD_4_-PDT could protect the integrity of the colonic mucosa, alleviate the inflammatory response and promote the healing of colonic mucosa. Mechanism studies demonstrated that LD_4_-PDT could inhibit the *NF-κB* signaling pathway, downregulated the expression of the inflammatory factors’ tumor necrosis factor-α (TNF-α), interleukin-6 (*IL-6*) and myeloperoxidase (*MPO*), increased the contents of glutathione (GSH) and superoxide dismutase (*SOD*) and decreased the content of malondialdehyde (MDA). Additionally, analysis of gut microbiota revealed that LD_4_-PDT treatment could decrease the abundance of the Proteobacteria phylum in fecal samples, while no significant differences were observed in the Firmicutes, Bacteroidetes, or Actinobacteria phyla among the three groups using 16S rRNA analysis.

**Discussion:**

In summary, our data suggested that LD_4_-PDT could inhibit DSS-induced UC in rats via the *NF-κB* signaling pathway, indicating its potential as a novel photosensitizer for the treatment of UC.

## Introduction

Ulcerative colitis (UC) is an inflammatory bowel disease characterized by inflammation and ulceration of the digestive tract (Ng et al., 2017). With the continuous improvement of living standards, the incidence of ulcerative colitis has been rising annually (Chow et al., 2009; Ng et al., 2017). At present, its etiology is not clear and may be related to environmental, infection, immunity, genetics and other factors (Costello et al., 2019). Existing therapeutic drugs include amino salicylic acid (Cevallos et al., 2021), glucocorticoids, immunosuppressants (Kang et al., 2021) or biological agents (Bröms et al., 2021), which primarily aim to suppress intestinal inflammation but often fall short in terms of safety and efficacy.

As research into the immune system deepens, therapeutic targets related to the pathogenesis of inflammation have been continuously discovered, leading to the development of various treatment modalities. Photodynamic therapy (PDT) is an innovative technique for disease diagnosis and treatment based on the photodynamic effect and is widely used in the treatment of various epidermal tumor diseases (Gunaydin et al., 2021). The advent of the endoscope and columnar optical fiber has enabled the application of PDT in the treatment of UC. Favre et al. (2011) reported that PDT in the treatment of UC with 5- aminoketone-pentanoic acid as a photosensitizer achieved good efficacy, while 5- aminoketone-pentanoic acid has no photosensitive activity, and it needs to be catalyzed into protoporphyrin Ⅸ to play a photosensitizer role, presenting challenges such as low concentration and uneven distribution (Maisch et al., 2011).

5,10,15,20-Tetra{4-[(S)-2,6-diamino-hexamide] phenyl} porphyrin (LD_4_) is a porphyrin synthesized in our laboratory, noted for its excellent water solubility and low toxicity (Meng et al., 2015). Previous studies have demonstrated that LD_4_-PDT promoted wound healing and immunomodulatory effects in the treatment of traumatic infection and exhibited good inhibitory and regulatory effects on microorganisms and pathogens (Xu et al., 2016; Zhao Y. et al., 2021). Additionally, We also confirmed it has the good treatment efficacy on UC caused by 2,4,6-trinitrobenzene sulfonic acid (Rong et al., 2021). Based on the above results, we speculated that photosensitizers have a therapeutic effect on DSS-induced UC. Therefore, the objective of this study was to prove the therapeutic effect of LD_4_-PDT on DSS-induced UC.

## Materials and methods

### Drugs and reagents

Our laboratory synthesized and characterized *LD*_*4*_ as previously described (Meng et al., 2015). The structure of LD_4_ is illustrated in Supplementary Figure S1A.

### Animals

Male Sprague-Dawley (SD) rats aged 6–8 weeks were purchased from Bei Jing HFK Bioscience Co., Ltd (SCXK 2019-0008; Beijing, China). All animal experimental procedures involving animals were performed according to the National Institutes of Health Guide for Care and Use of Laboratory Animal Management Committee/Laboratory Animal Welfare Ethics Committee, Institute of Radiation Medicine, Chinese Academy of Medical Sciences (Approval No. IRM-DWLL-2023196). The rats were reared under standardized conditions, with an ambient temperature of 22°C ± 2°C and relative humidity maintained at 40%–70%. They had *ad libitum* access to food and water. All procedures involving the rats adhered to the guidelines established in the National Institutes of Health’s Laboratory Animal Care and Use Guidelines.

### Building model of dextran sodium sulfate (DSS)-induced UC and corresponding treatment

The rats were evenly divided into six groups, each comprising of 6 individuals, and were subjected to the following treatments: (1) control group (normal saline); (2) 3%DSS group (3%DSS); (3) LD_4_ photodynamic therapy low dose (LD_4_-PDTL) group (3%DSS and low-dose LD_4_ [60 μg/kg]; (4) LD_4_ photodynamic therapy medium dose (LD_4_-PDTM) group (3%DSS and medium-dose LD_4_ [120 μg/kg]); (5) LD_4_ photodynamic therapy high dose (LD_4_-PDTH) group (3%DSS and high-dose LD_4_ [240 μg/kg]); (6) Sulfasalazine (SASP) group (3%DSS and sulfasalazine [SASP, 500 mg/kg]). The rats in the experimental groups were treated with both 3% DSS and drugs. The administration of 3% DSS in drinking water was recorded as day 0, and treatment commenced on the 7th day, as detailed in Supplementary Figure S1B. LD_4_ was administered every other day for a total of four treatments. The photosensitizer was given by enema inside the colon before irradiation. 30 min later, the colon was irradiated by a 650 nm semiconductor laser (WSLS-650-500 M-200 M-H4; China) at an energy density of 25 J/cm^2^ via a fiber coated a rubber pipe to protect the colon from thermal burn in each treatment. When all four treatments were completed, all rats were sacrificed, and whole colons and blood were collected after 24 h of fasting. Partial colon tissues were fixed with 4% paraformaldehyde for subsequent experiment.

### FITC-dextran fluorescence intensity test

The degree of intestinal permeability in rats was determined by detecting the fluorescence intensity in serum. Two hundred micrograms of FITC-dextran (FD40S; Sigma–Aldrich) powder was dissolved in 5 mL rat serum and diluted at various ratios to generate a standard curve, and then the fluorescence intensity was detected with an enzyme plate analyzer (Thermo Fisher, MA) to obtain the standard curve. After all the treatment, the animals were fasted for 4 h before they were sacrificed. The prepared FITC-dextran tracer was given by gavage at a dose of 0.6 mg/g. Serum samples, collected from rats without hemolysis, were placed in a 96-well plate at 100 μL per well. The fluorescence intensity was measured (Ex/Em: 488/520 nm), and the concentration of FITC-dextran in rat serum was determined using the established standard curve.

### Biochemical analysis

Interleukin-6 *(IL-6)* (SEA079Ra; Cloud-Clone Corp) and tumor necrosis factor-α (*TNF-α*) (SEA133Ra; Cloud-Clone Corp) were obtained from Cloud-Clone Corp, China and quantified using ELISA kits. The levels of myeloperoxidase (*MPO*) (A044-1-1), glutathione (*GSH*) (A006-1-1), Malondialdehyde (MDA) (A003-1-2) and Superoxide dismutase (SOD) (A001-1-2)in the serum of rats were purchased from Nanjing Jian Cheng Institute of Biological Engineering, China.

### Histopathological analysis

Colon tissue was sectioned into 5 μm slices for histopathological analysis. The hematoxylin-eosin (HE) staining procedure was conducted as previously described (Liu et al., 2016), and the degree of inflammation was scored in accordance with the methodology outlined in the literature (Rong et al., 2018).

### Western blotting analysis

The method of protein extraction was described in our previous article (Rong et al., 2018). Antibodies were diluted with TBST and configured according to the instructions. Rabbit anti-*p-NF-κB* (3033S; United States), *NF-κB* (82425; United States), p-IκB (2859S; United States), *IκB* (4812; United States), *p-IKKα/β* (ab178870; United States) antibodies were purchased from Abcam; rabbit anti-*IL-6* (WL02841; China) and *TNF-α* (WL01581; China) monoclonal antibodies were purchased from Wanleibio.

### Molecular docking study

Molecular docking was performed using the Glide module of the Schrödinger suite 2009. The optimal protein structure (3HIG) of AOC1 was retrieved from the PDB protein database. The protein underwent pretreatment, including review, modification, and refinement. Following the addition of hydrogen atoms and removal of all bound water molecules, protein optimization was conducted using a redirected hydrogen bond network, with default parameters selected for the docking calculations.

### Bacterial diversity analysis

As the treatment was completed, feces from nine rats were collected in different cages, and DNA was extracted from the fecal samples. The concentration of DNA in the library was determined by a Qubit Fluorometer, and greater than 1.0 ng/μL was considered qualified sample. The Illumina MiSeq sequencing platform was used to amplify and sequence the V3–V4 region of the bacterial 16S ribosomal RNA (rRNA) gene. The data were analyzed by USEARCH (http://www.drive5.com/usearch/7.0). Bioinformatics analysis was performed according to the operational taxonomic unit (OTU). Sequence similarity greater than 97% was classified as an operational taxonomic unit (OUT). The bacterial 16S rRNA forward primer sequence was 5′-CCTACGGGNGGCWGCAG-3′, and the reverse primer sequence was 5′-GACTACHVGGGTATCTAATCC-3′.

### Statistical analysis

Data analysis was conducted using Graph Pad Prism 7 Software. All measurement data are expressed as the mean ± standard deviation. Statistical significance was evaluated by using one-way analysis of variance. *P* < 0.05 was considered statistically significant.

## Results

### LD_4_-PDT ameliorated colon inflammation protected the integrity of the intestine in DSS-induced UC rats

**FIGURE 1:**
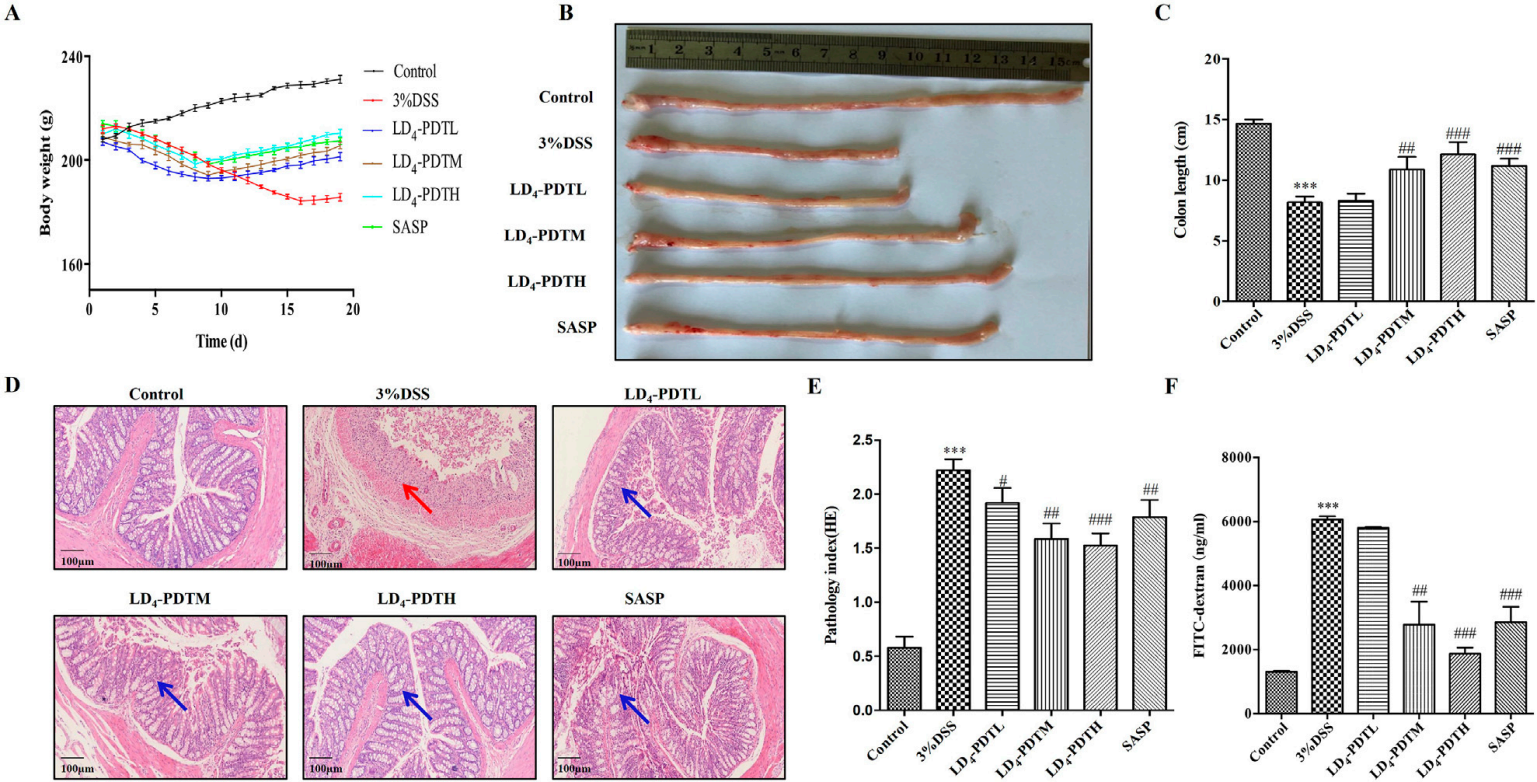
LD_4_-PDT ameliorated colon inflammation in DSS-induced UC model rats. **(A)** The body weight. **(B, C)** The length of colon and analysis. **(D)** H&E staining of colon tissue and **(E)** inflammation score; **(F)** Fluorescence intensity of FITC-dextran in rat serum; n = 3–6 rats per group. ^***^ *P* < 0.001 vs. the control group; ^*#*^ *P* < 0.05, ^##^ *P* < 0.01, ^###^ *P* < 0.001 vs. the 3% DSS model group.

As illustrated in Figure 1A, the average body weight of rats in the control group increased steadily, while the body weights in the SASP and LD_4_-PDT treatment groups also exhibited gradual increases. In contrast, the body weight of rats in the 3% DSS model group decreased significantly. Otably, the three different drug concentrations exhibited dose-dependent effects. As shown in Figures 1B, C, compared with the control group, the colon wall of rats in the 3% DSS group was thicker and shorter, while the length of colon tissue in the LD_4_-PDTM, LD_4_-PDTH and SASP groups protected the colon from DSS-induced damage. As shown in Figures 1D, E demonstrated that colon epithelial cells in the control group were neatly arranged, with no infiltration of inflammatory cells and abundant goblet cells observed. Conversely, the 3% DSS group exhibited various pathological features, including epithelial cell loss, multiple colonic ulcers, infiltration of inflammatory cells into the mucosa and submucosa, and a reduction in goblet cells. In comparison, the colonic surface mucosa of rats in the SASP group and all LD_4_-PDT dosage groups showed substantial recovery, with an increase in goblet cells, effective healing of ulcers, and a significant decrease in neutrophil infiltration in both the mucosa and submucosa. Notably, the three different drug concentrations exhibited dose-dependent effects.

FITC-dextran, a fluorescent dye, was utilized to assess intestinal permeability, with fluorescence intensity in serum serving as an indicator of intestinal integrity. As depicted in Figure 1F, the FITC-dextran content in the control group was low, indicating normal intestinal permeability. In contrast, the FITC-dextran content in the 3% DSS group was significantly elevated, reflecting increased intestinal permeability and compromised intestinal wall integrity. Comparatively, the FITC-dextran content in the LD_4_-PDT group showed a marked decrease in a dose-dependent manner across low, medium, and high doses, indicating that LD_4_-PDT provides protective effects on intestinal integrity.

### LD_4_-PDT decreased the expression of inflammatory cytokines in DSS-induced UC rats

**FIGURE 2:**
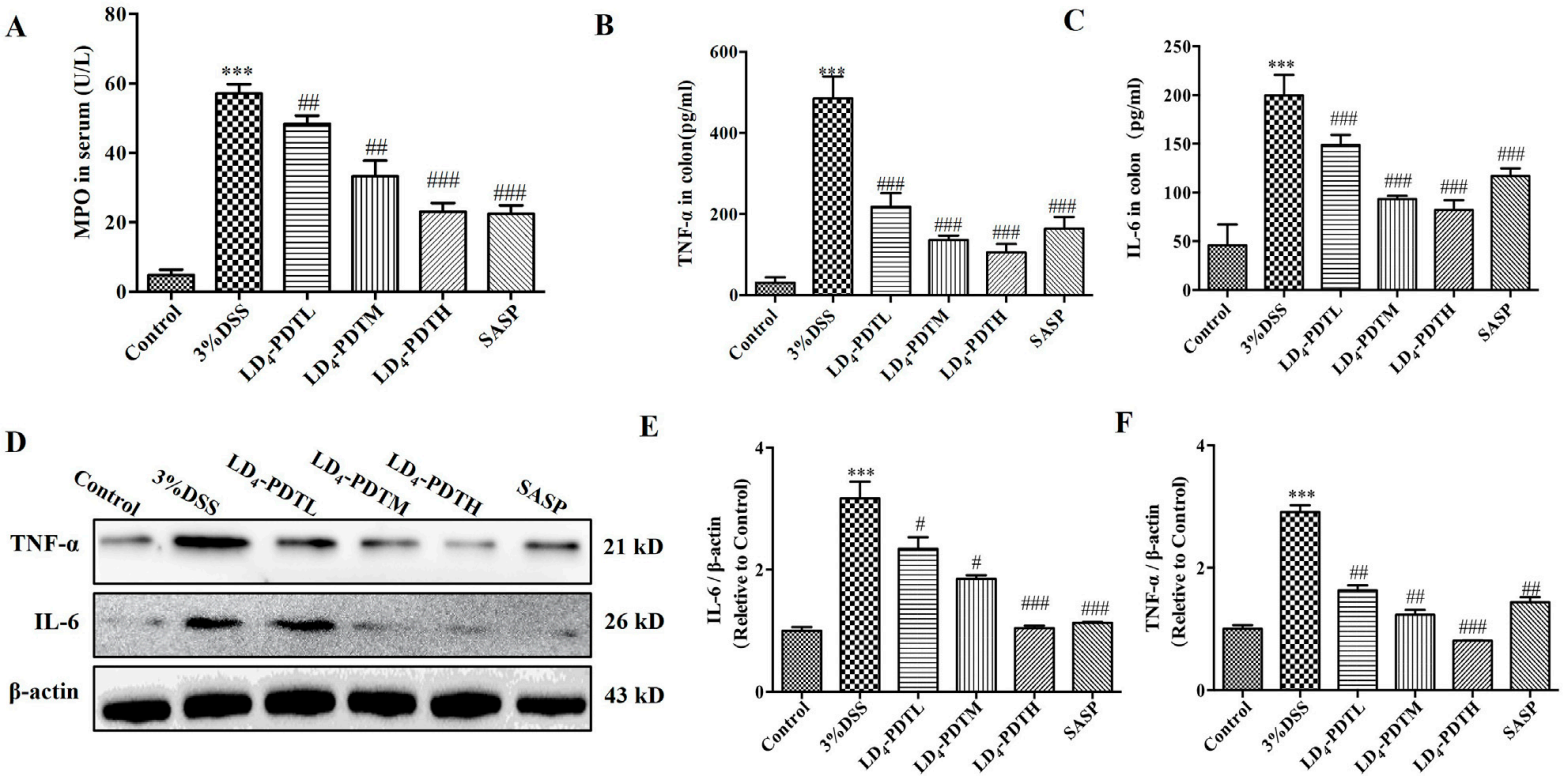
LD_4_-PDT decreased the expression of inflammatory cytokines in DSS-induced UC rats. **(A)** The content of *MPO* in serum. **(B, C)** *IL-6* and *TNF-α* levels were tested by ELISA. **(D)** Protein expression of **(E)** *IL-6* and **(F)** *TNF-α* in colon tissue were determined by Western blot. Each column expresses the mean ± SD of 3-6 rats per group. ^***^ *P* < 0.001 vs. the control group; ^#^ *P* < 0.05, ^##^ *P* < 0.01, ^###^ *P* < 0.001 vs. the 3% DSS model group.

The levels of various inflammatory cytokines, including myeloperoxidase (MPO), interleukin-6 (IL-6), and tumor necrosis factor-alpha (TNF-α), were further evaluated. As shown in Figures 2A–C, the concentrations of *MPO*, *IL-6* and *TNF-α* in the serum or colon tissue from control group rat were lower, while these data were significantly increased in the 3% DSS group, and were significantly decreased in the SASP and LD_4_-PDT groups, and three types of drug concentrations exhibit dose-dependent effects. Additionally, consistent results were obtained from the Western blot analysis, as shown in Figure 2D, which indicated reduced protein expression levels of *IL-6* and *TNF-α* in the colon tissues of the treatment groups.

### LD_4_-PDT attenuated oxidative stress in DSS-induced UC rats

**FIGURE 3:**
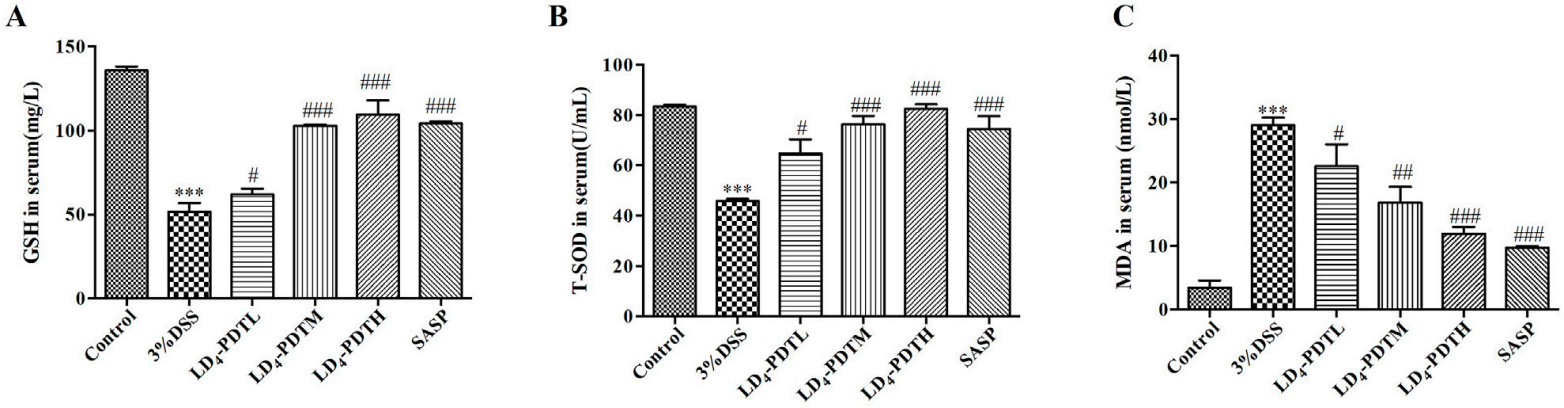
LD_4_-PDT regulated oxidative stress in DSS-induced UC rats. **(A–C)** *GSH*, *SOD* and *MDA* levels in rat serum were measured by ELISA. Each column expresses the mean ± SD of 3-6 rats per group. ^***^ *P* < 0.001 vs. the control group; ^#^ *P* < 0.05, ^##^ *P* < 0.01, ^###^ *P* < 0.001 vs. the 3% DSS model group.

Oxidative stress plays an important role in the occurrence of UC (Linares et al., 2011). MDA is one of the main products of lipid peroxidation and is commonly used as an indicator of oxidative stress. The accumulation of MDA can cause certain damage to cell membranes and organelles. Testing the amount of malondialdehyde (MDA) can reflect the degree of lipid peroxidation in the body, indirectly reflecting the degree of cell damage. GSH and SOD have antioxidant effects, protecting cells from oxidative damage. We further detected some redox factors, including *GSH*, *SOD* and *MDA*. As shown in Figures 3A–C, the levels of two antioxidants in rat serum, *GSH* and *SOD*, were higher in the control group, were significantly reduced in the 3% DSS group, and were increased significantly in the SASP and LD_4_-PDT groups. The *MDA* content was significantly increased in the 3% DSS group but was significantly decreased in the SASP- and LD_4_-PDT-treated rats. We observed that three types of drug concentrations exhibit dose-dependent effects. Collectively, these findings suggest that LD_4_-PDT effectively attenuates oxidative stress in DSS-induced UC rats.

### LD_4_-PDT suppressed the NF-κB signaling pathway in DSS-induced UC rats

**FIGURE 4:**
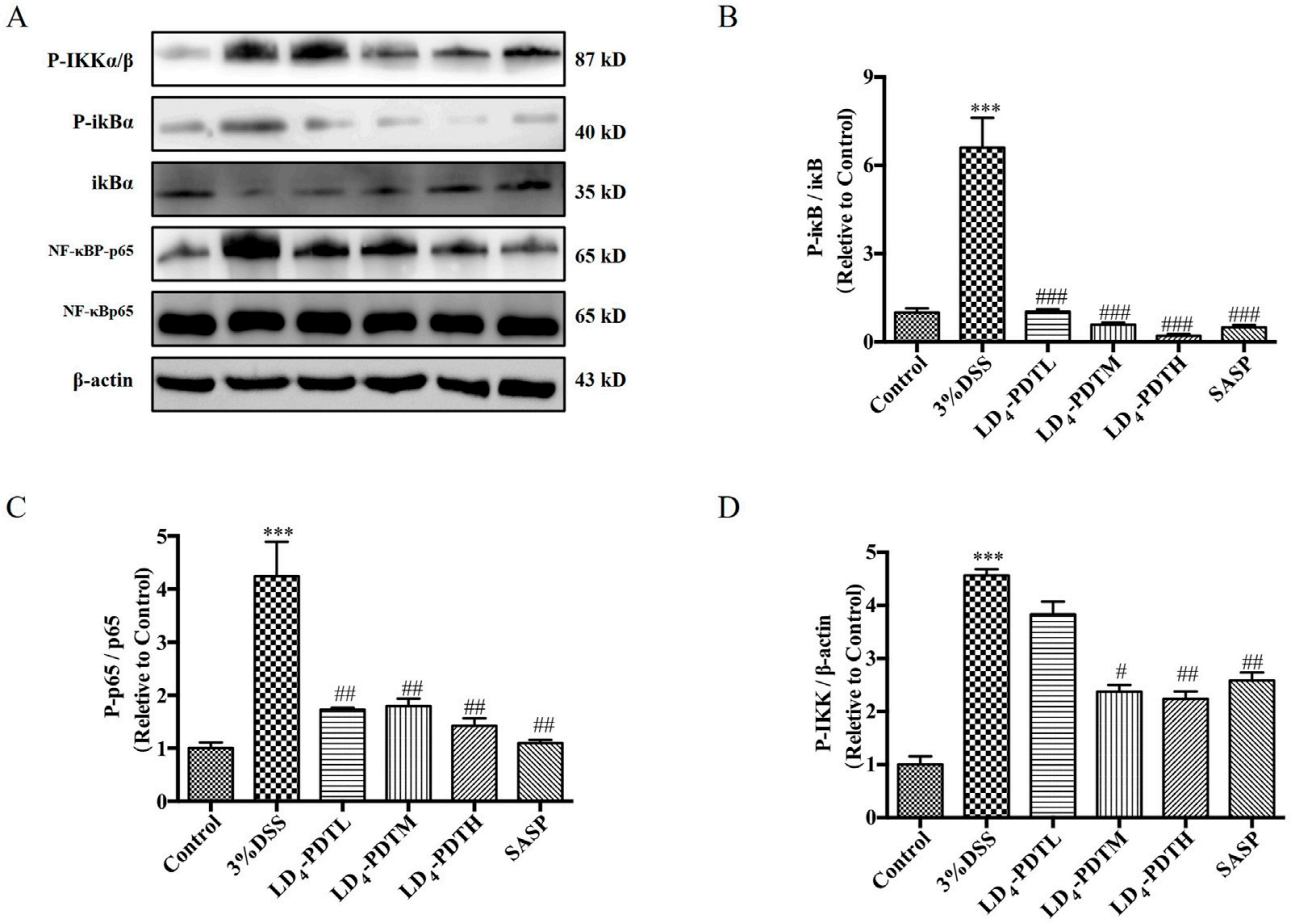
LD_4_-PDT suppressed the NF-κB signaling pathway in DSS-induced UC model rats. **(A)** The expression of **(B)** *p-IκB*, *IκB*, **(C)** *NF-κBP-p65* and *NF-κBp65* **(D)** *p-IKK* in colon tissue was measured by Western blot. ^***^ *P* < 0.001 vs. the control group; ^#^ *P* < 0.05, ^##^ *P* < 0.01, ^###^ *P* < 0.001 vs. the 3% DSS model group; n = 3 rats per group. Data were presented as the means ± SD based on three independent experiments.

Studies have shown that activation of the *NF-κB* signaling pathway plays a key role in both inflammation and UC (Pervin et al., 2016; Mohamed and Said, 2021). Therefore, we detected the effect of LD_4_-PDT on the *NF-κB* signaling pathway by Western blotting. As shown in Figures 4A–D, compared with the control group, the expression of *p-IKK, p-IκB* and *p*-*NF-κB* was increased significantly in the 3% DSS group, while significantly decreased in SASP and each dose group of LD_4_-PDT treatment, indicated that LD_4_-PDT could suppress the *NF-κB* signaling pathway in DSS-induced UC rats.

### Molecular docking study

**FIGURE 5:**
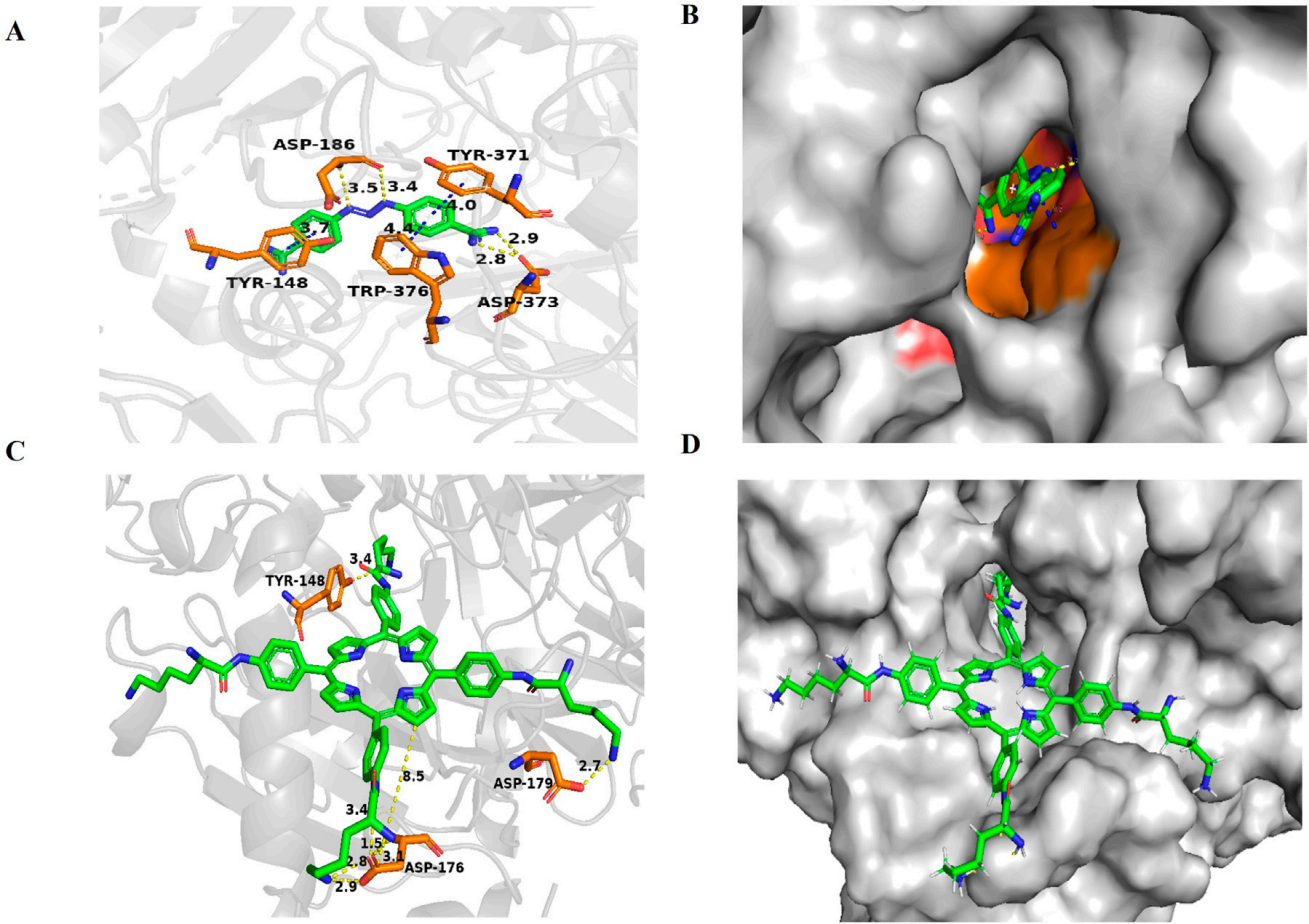
Docking and binding pattern into AOC1 active site for ligand **(A, B)** and LD_4_ **(C, D)**. Hydrogen bonds - yellow lines, π-π bonds - blue lines.

In previous research, to further explore the biological mechanism of LD_4_-PDT in UC, colonic protein assays were conducted using proteomics in model, control and LD_4_-PDT groups. More than 176 proteins were detected and their differential expression were given between model and LD_4_-PDT group. *AOC1* was abundance changed and correlated tightly with the inflammation, was identified as a potential target of LD_4_-PDT (Rong et al., 2021). To further illustrate, we did molecular docking. To explore the binding patterns of LD_4_ and *AOC1*, the optimal 3HIG structure of the *AOC1* protein selected from the PDB protein structure database was maleducative docking using Schrödinger suite 2009. As shown in Figures 5A–D the results of LD_4_ were embedded in the binding pocket of 3HIG protein and bound to the active binding site of 3HIG protein via H bond (yellow), π-π bond (blue), and amino acid on the protein (orange). The binding mode diagram explains the effect of LD_4_ on the activity of *AOC1*. Binding energy is released when a particle is combined from a free state into a composite particle. The smaller the binding energy value, the more stable the molecular structure. The lower the absolute value of the docking score, the better the binding effect. The ligand structure is depicted in Supplementary Figure S3. As can be seen from Supplementary Table S1, the docking fraction and absolute binding energy of LD4 were found to be superior to those of the ligand, suggesting that LD4 interacts more closely with AOC1, exhibiting a stronger binding effect.

### LD_4_-PDT regulated gut bacterial composition in DSS-induced UC model rats

**FIGURE 6:**
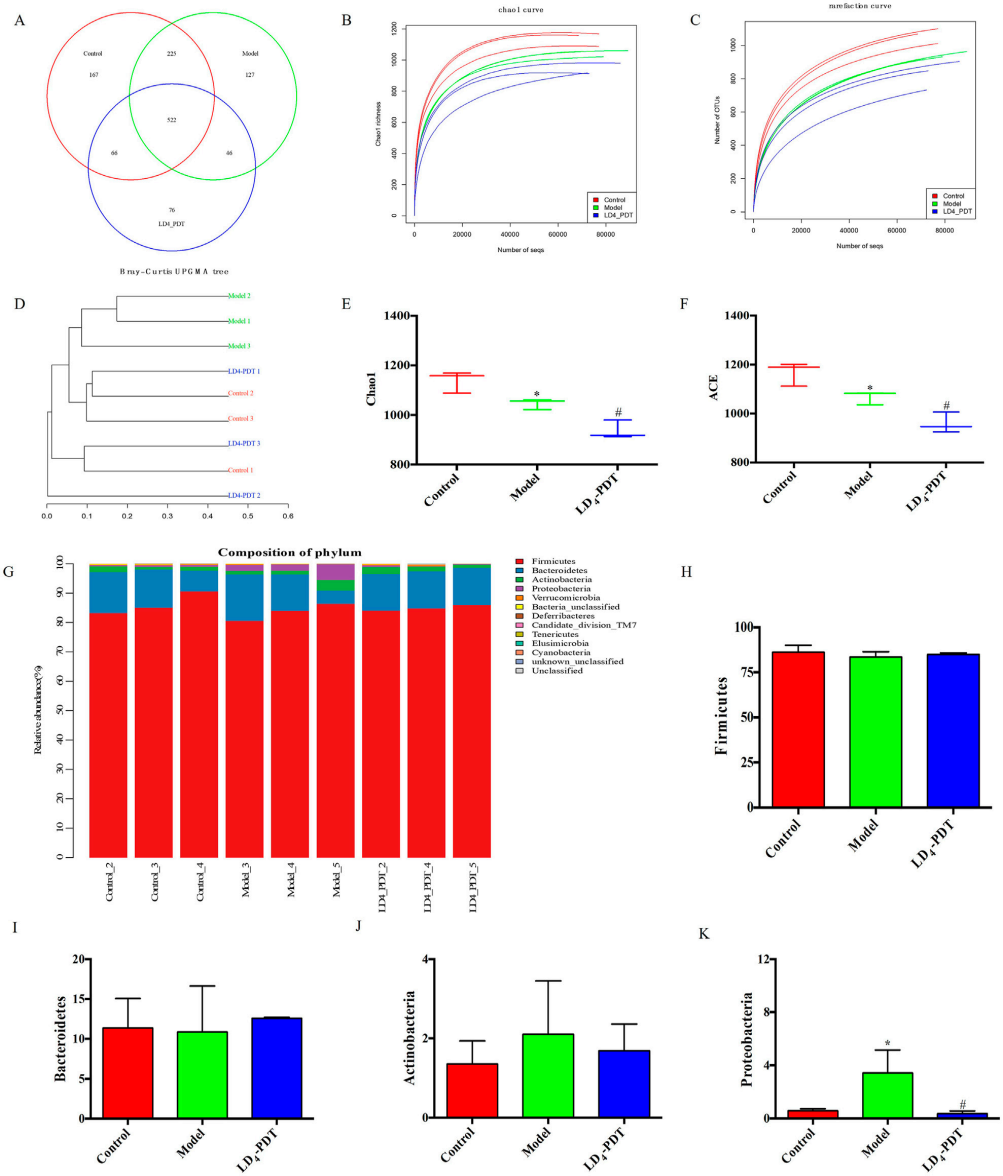
LD_4_-PDT regulated gut bacterial composition in DSS-induced UC model rats. **(A)** Venn diagram of OTUs in the three groups. **(B)** Chao1 curve. **(C)** Rare faction curve. The horizontal axis is the number of sequences, and the vertical axis is the diversity index. **(D)** Bray−Curtis UPGMA tree of all sample. **(E)** Chao1 index. **(F)** The ACE index of intestinal bacteria in rats. **(G)** Microbial composition bar plot by phylum. **(H–K)** Relative abundance of **(H)** Firmicutes, **(I)** Bacteroidetes, **(J)** Actinobacteria, and **(K)** Proteobacteria. ^*^ *P* < 0.05 vs. the control group; ^#^ *P* < 0.05 vs. the model group; n = 3 rats per group.

The changes in intestinal microflora among the control group, 3% DSS group and LD_4_-PDT group were examined by using the 16S rRNA method. The Venn diagram showed that a total of 1,229 OTUs were obtained from all samples when the similarity was 97%. A total of 522 OTUs were detected in the three groups. 66 OTUs were detected in the control group and LD_4_-PDT group, and 225 OTUs overlapped between the 3% DSS group and the control group. The common OTU of the 3% DSS group and the LD_4_-PDT group was 76 (Figure 6A). The Chao1 curve and rarefaction curve showed that alpha diversity in the LD_4_-PDT group was lower than those in the control group and 3% DSS group (Figures 6B, C). UPGMA analysis showed that there was a significant separation of intestinal flora between the 3% DSS group and the LD_4_-PDT group. (Figure 6D). The α-diversity of intestinal bacteria in 3% DSS group decreased significantly compared with the control group, while LD_4_-PDT treatment significantly decreased the diversity (Figures 6E, F). There was no significant difference between the Firmicutes, Bacteroidetes and Actinobacteria phyla in the three treatment groups (Figures 6G–J). Compared with the control group, 3% DSS increased the Proteobacteria phylum in fecal stool samples, while LD_4_-PDT treatment decreased the Proteobacteria phylum in fecal stool samples (Figure 6K). In conclusion, our study demonstrated that LD_4_-PDT regulated gut bacterial composition in DSS-induced UC model rats.

## Discussion

UC is one of the most prevalent and challenging conditions to treat within the field of clinical gastroenterology (Morris et al., 1989). Exploring an ideal treatment is the goal of the research. The choice of model is the key to the success of the experiment. DSS-induced UC has a direct toxic effect on surface epithelial cells, causing acute colitis that is similar to human morphology and symptoms (Song et al., 2015). The advantage of this model is that it is of great significance to the pathogenesis, etiology and drug development of UC (Zhang et al., 2018). Following the establishment of the model, the colonic mucosal barrier is compromised, leading to inflammatory cell infiltration. Treatment with LD_4_-PDT has been shown to mitigate inflammatory infiltration, promote mucosal healing, and preserve intestinal integrity.

Many studies have shown that proinflammatory cytokines also play an important role in the occurrence and development of UC (Chu et al., 2015). Neutrophils contain a proteolytic enzyme called *MPO*, which has an antibacterial effect (Gupta et al., 2015). *TNF-α* is a tumor necrosis factor that can promote T cells to produce various inflammatory factors and promote the occurrence of the inflammatory response (Zhao Z. et al., 2021). Similarly, *IL-6* can regulate the growth and differentiation of a variety of cells and the immune response and plays an important role in the body’s anti-infection immune response (Zhu et al., 2019). Studies (Magalhães et al., 2021; Zhao Z. et al., 2021) demonstrated elevated levels of *MPO*, *TNF-α* and *IL-6* in UC model mice, and corresponding inhibition of their secretion can reduce the severity of UC. In our study, LD_4_-PDT treatment significantly reduced the levels of *MPO*, *TNF-α*, and *IL-6* and then reduced the degree of inflammation in UC.

*SOD* can eliminate harmful substances produced in the process of metabolism, and *GSH* can remove the structural and functional integrity of cell membranes protected by reactive oxygen species. However, *MDA* can cause the cross-linked polymerization of living macromolecules such as proteins and nucleic acids, and it is cytotoxic (Li et al., 2017). After the induction of colitis, oxidative stress will occur in the body, and the expression of *MDA* in the colon tissue is increased, while the expression of *SOD* and *GSH* is decreased. LD_4_-PDT treatment significantly reduced the *MDA* content, increased the *SOD* and *GSH* contents, and then regulated oxidative stress to protect the intestinal tract.

**FIGURE 7:**
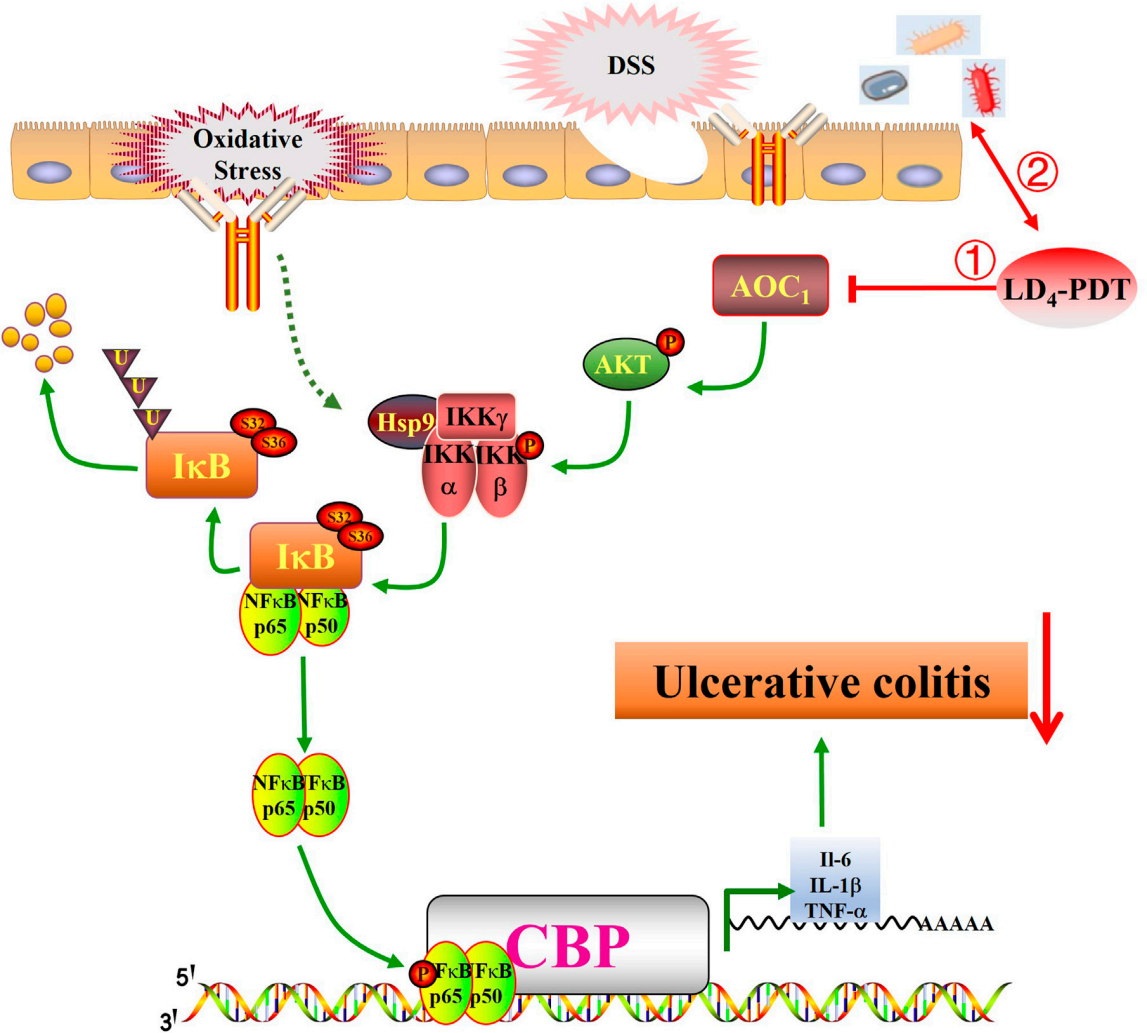
LD_4_-PDT inhibits DSS-induced ulcerative colitis in rats via the NF-κB signaling pathway. LD_4_-PDT reduces AOC1-mediated mucosal inflammation, promotes the healing of colon mucosa, regulates intestinal flora, and improves the clinical symptoms of UC.

The human gut microbes is home to a diverse community of microorganisms, including bacteria, viruses, fungi, and protozoa. These microbes coexist in a symbiotic relationship with their host, providing various benefits such as aiding in digestion, synthesizing vitamins, and modulating the immune system. In health, the gut microbiota is diverse and stable, but in IBD, this balance is disrupted. Some studies have reported an increase in the phylum Firmicutes and a decrease in Bacteroidetes in UC model groups. The delicate balance of gut microbes is crucial for maintaining health, and disruptions to this equilibrium can lead to various diseases, including inflammatory bowel disease (IBD). IBD, which encompasses conditions like ulcerative colitis (UC) and Crohn’s disease, is characterized by chronic inflammation of the gastrointestinal tract. As shown in Figure 7, following UC model establishment, the *AKT/IKK/NF-κB* pathway is activated, promoting the occurrence and development of inflammatory responses. Following LD_4_-PDT treatment, the *AKT/IKK/NF-κB* pathway is suppressed, thereby exerting a therapeutic effect on UC. The mechanism of action may involve LD_4_-PDT mediating the expression of the *AKT/IKK/NF-κB* pathway and downstream inflammatory cytokines through *AOC1*. Consequently, LD_4_ has the potential to serve as a novel photosensitizer in the treatment of ulcerative colitis. The exact etiology of IBD is complex and multifactorial, involving genetic, environmental, and immunological factors. However, it is increasingly recognized that alterations in the gut microbiota play a significant role in the pathogenesis of IBD (Li et al., 2020). Some studies have shown that Firmicutes was increased and Bacteroidetes was decreased in the UC model group (Shan et al., 2018; Zakerska-Banaszak et al., 2021), suggesting a shift in the microbial composition that may contribute to disease development. Conversely, other studies have found no significant difference in Firmicutes and Bacteroidetes between UC patients and healthy controls (Nishino et al., 2018), indicating that the relationship between these phyla and IBD may be more complex than initially thought.

Our study contributes to this understanding by revealing that in rats, Firmicutes and Bacteroidetes are the most abundant bacteria, followed by Actinobacteria and Proteobacteria. The presence of Proteobacteria, a phylum that includes many pathogenic bacteria, has been linked to microecological dysregulation and an unstable gut microbiome structure. It is often overrepresented in intestinal and extraintestinal diseases with inflammatory phenotypes (Shin et al., 2015; Litvak et al., 2017). The restoration of a healthy gut microbiota through treatments like LD_4_-PDT may therefore provide a novel therapeutic approach for IBD. By reducing the levels of potentially pathogenic bacteria and promoting a more balanced microbial community, such treatments could help to reestablish a stable gut environment, reduce inflammation, and alleviate IBD symptoms. Future research should focus on understanding the specific mechanisms by which LD_4_-PDT and other interventions modulate the gut microbiota and influence IBD pathogenesis. This could lead to the development of targeted therapies that harness the power of the microbiota to treat and prevent IBD.

Inflammatory factors, oxidative stress and bacterial viruses can stimulate and activate the *NF-κB* pathway, so inhibition of the *NF-κB* signaling pathway may be the treatment approach for UC (Pervin et al., 2016; Zhu et al., 2019; Yu et al., 2020). Normally, *NF-κB* is composed of a *p65/p50* heterodimer and inactive in the cytoplasm. When the upstream kinase activates the *IKK* complex, it promotes the phosphorylation of IκB, leading to its degradation and subsequent release of the *p65/p50* heterodimer, and the heterodimer is rapidly transferred into the nucleus to activate the transcription of various inflammatory mediators (Perkins, 2007). Our study showed that LD_4_-PDT treatment significantly reduced the expression of *p-IKK, p-IκB, and p-NF-κB* and increased the expression of *IκB*, so LD_4_-PDT can inhibit the *NF-κB* signaling pathway to treat UC. Many studies have proven that PDT can realize the treatment of UC, but the safety of photosensitizers needs to be further evaluated. Further clinical trials are needed to evaluate its efficacy and safety for the enema with large doses of LD_4_. Following UC model establishment, the *AKT/IKK/NF-κB* pathway is activated, promoting the occurrence and development of inflammatory responses. Following LD_4_-PDT treatment, the *AKT/IKK/NF-κB* pathway is suppressed, thereby exerting a therapeutic effect on UC. The mechanism of action may involve LD_4_-PDT mediating the expression of the *AKT/IKK/NF-κB* pathway and downstream inflammatory cytokines through *AOC1*. Consequently, LD_4_ has the potential to serve as a novel photosensitizer in the treatment of ulcerative colitis.

## Data Availability

The datasets presented in this study can be found in online repositories. The names of the repository/repositories and accession number(s) can be found in the article/Supplementary Material.
