# Supplementary material for: Photodynamic therapy with a novel photosensitizer inhibits DSS-induced ulcerative colitis in rats via the NF-κB signaling pathway
Source: Front Pharmacol. 2025 Jan 8;15:1539363. doi: 10.3389/fphar.2024.1539363 (PMC11750845; doi:10.3389/fphar.2024.1539363)
Supplement: Supplementary file 1 [file DataSheet1.docx]

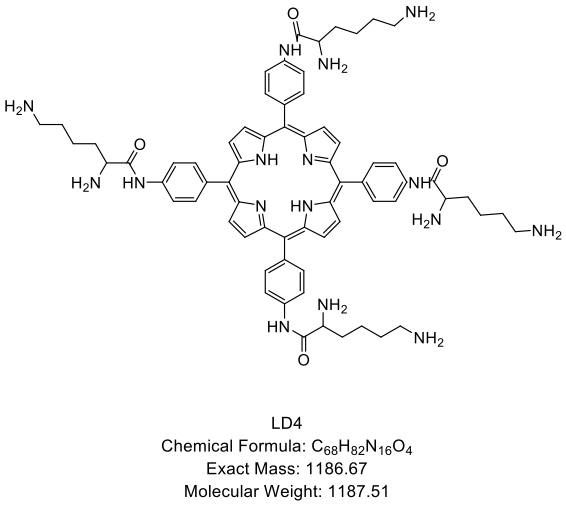
Photodynamic therapy with a novel photosensitizer inhibits DSS-induced ulcerative colitis in rats via the NF-κB signaling pathway

**Fig1**

**A**

A

**5,10,15,20-Tetra{4-[(S)-2,6-diamino-hexamide] phenyl} porphyrin (LD_4_)**

B


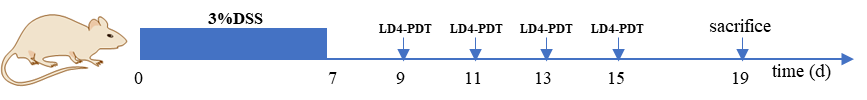


The rats for experimental groups were treated with both 3% DSS and drugs. The first day on which giving rats 3% DSS in drinking water was recorded as day 0, on the 7th day treatment was initiated, LD_4_ was administered every second day via enema for a total of four treatments. On the 19th day, rats were sacrificed for tissue collection and analysis.

C


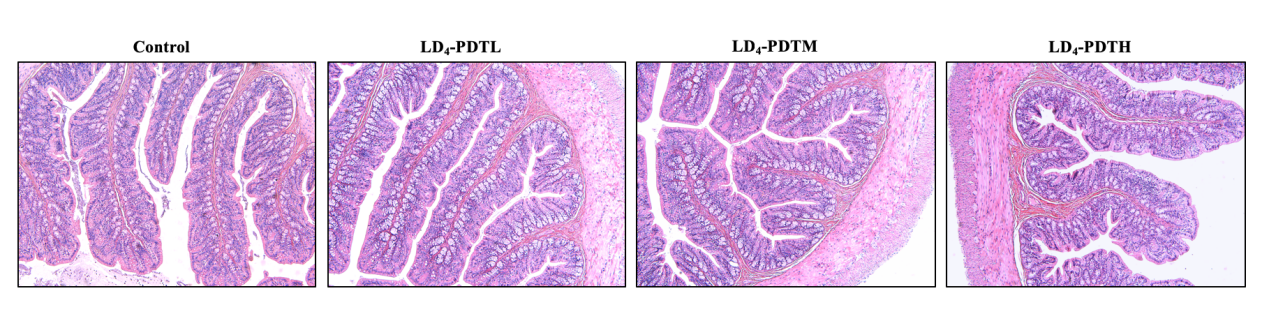


Effects of three different doses of LD_4_ on normal rats

Fig2 The Western Blot results of all rats


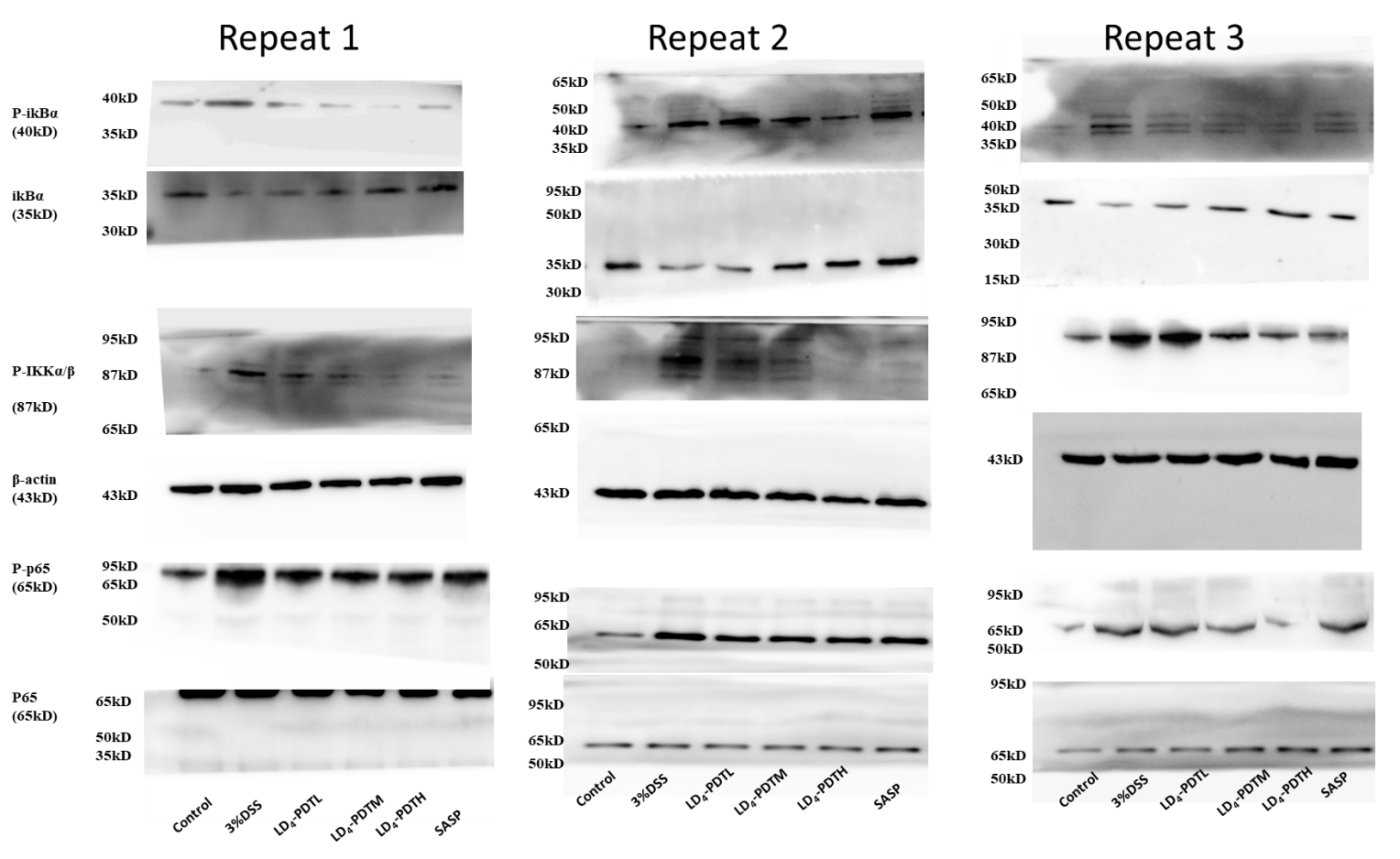


Fig 3


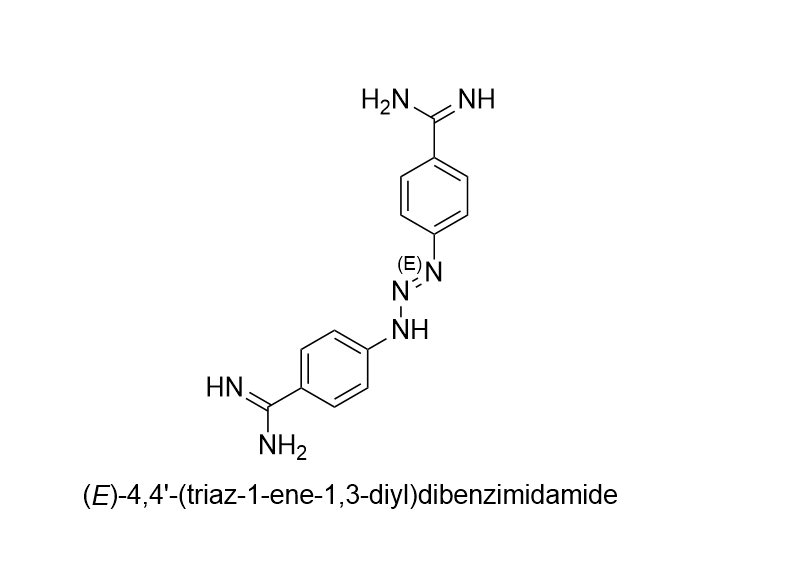


**Supplementary Tables**

**Supplementary Table S1**

Table 1 Molecular docking tuberculosis energy

| Compound | Docking score(kcal/mol) | | Binding energy(kcal/mol) | |
| --- | --- | --- | --- | --- |
| Ligand | | -11.485 | | -57.210 |
| LD_4_ | | -14.504 | | -102.827 |

**
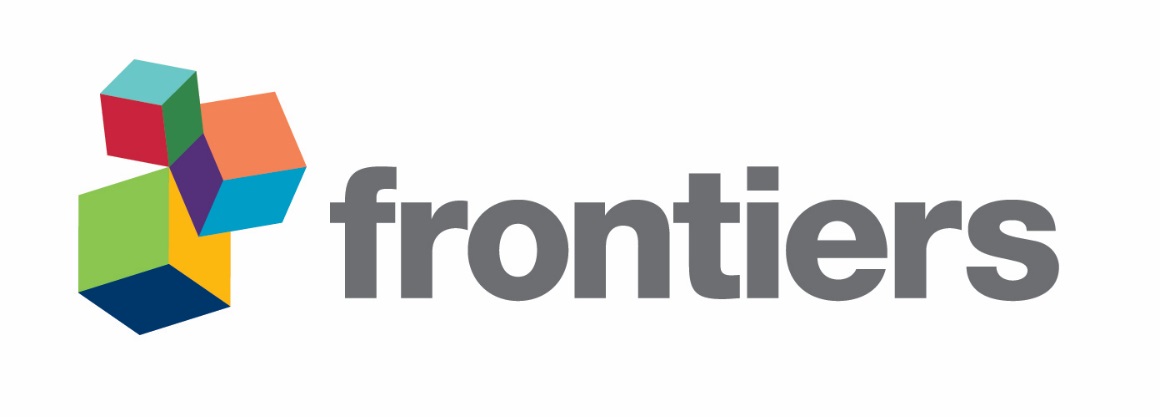
**
